# Human artificial chromosome carrying 3p21.3-p22.2 region suppresses *hTERT* transcription in oral cancer cells

**DOI:** 10.1007/s10577-023-09726-8

**Published:** 2023-06-24

**Authors:** Takahito Ohira, Kaho Yoshimura, Hiroyuki Kugoh

**Affiliations:** 1 Department of Chromosome Biomedical Engineering, Tottori University, 86 Nishi-Cho, Yonago, Tottori 683-8503 Japan; 2 Chromosome Engineering Research Center, Tottori University, 86 Nishi-Cho, Yonago, Tottori 683-8503 Japan; 3 Division of Genome and Cellular Function, Department of Molecular and Cellular Biology, Tottori University, 86 Nishi-Cho, Yonago, Tottori 683-8503 Japan

**Keywords:** Chromosome engineering, Tumor suppressor, Telomerase, Human telomerase reverse transcriptase (*hTERT*), Oral squamous cell carcinoma

## Abstract

Telomerase is a ribonucleoprotein ribonucleic enzyme that elongates telomere repeat sequences at the ends of chromosomes and contributes to cellular immortalization. The catalytic component of telomerase, human telomerase reverse transcriptase (*hTERT*), has been observed to be reactivated in immortalized cells. Notably, most cancer cells have been found to have active *hTERT* mRNA transcription, resulting in continuous cell division, which is crucial for malignant transformation. Therefore, discovering mechanisms underlying the regulation of *hTERT* transcription is an attractive target for cancer-specific treatments.

Loss of heterozygosity (LOH) of chromosome 3p21.3 has been frequently observed in human oral squamous cell carcinoma (OSCC). Moreover, we previously reported that HSC3 OSCC microcell hybrid clones with an introduced human chromosome 3 (HSC3#3) showed inhibition of *hTERT* transcription compared with the parental HSC3 cells. This study examined whether *hTERT* transcription regulators are present in the 3p21.3 region. We constructed a human artificial chromosome (HAC) vector (3p21.3-HAC) with only the 3p21.3-p22.2 region and performed functional analysis using the 3p21.3-HAC. HSC3 microcell hybrid clones with an introduced 3p21.3-HAC exhibited significant suppression of *hTERT* transcription, similar to the microcell hybrid clones with an intact chromosome 3. In contrast, HSC3 clones with truncated chromosome 3 with deletion of the 3p21.3 region (3delp21.3) showed no effect on *hTERT* expression levels. These results provide direct evidence that *hTERT* suppressor gene(s) were retained in the 3p21.3 region, suggesting that the presence of regulatory factors that control telomerase enzyme activity may be involved in the development of OSCC.

**Supplementary Information:**

The online version contains supplementary material available at 10.1007/s10577-023-09726-8.

## Introduction

Immortalization is a key component of cancer phenotypes. Telomere length is known to shorten with cell replication in normal somatic cells. This progressive shortening is strongly associated with cellular senescence and apoptosis (Bernardes de Jesus and Blasco 2013). In contrast, in most human cancer cells, telomere length is maintained by telomerase. Thus, telomere length and telomerase activity are crucial for cancer initiation and survival. The human telomerase reverse transcriptase (*hTERT*) catalytic subunit mainly regulates telomerase activity. Indeed, ectopic *hTERT* expression in telomerase-negative normal cells can extend the lifespan and establish immortalized cell lines via telomere elongation (Piao et al. 2005).

Oncogenes and tumor suppressor genes, which are involved in activating and inhibiting cell proliferation, respectively, play critical roles in cancer development. The concept of a tumor suppressor gene was proposed based on cytogenetic and molecular biology analyses of whole cell–cell fusion of normal and cancer cells and loss of heterozygosity (LOH) (Kugoh et al. 2015). We previously reported that the introduction of normal human chromosome 3 using microcell-mediated chromosome transfer (MMCT) induced cellular senescence in two immortal renal cell carcinoma (RCC) cell lines, RCC23 and KC12, which have LOH on the short arm of chromosome 3 (Abe et al. 2010; Tanaka et al. 1998). A reduction in *hTERT* expression and telomerase activity accompanies this phenomenon. Furthermore, we found a telomerase repression gene within a 7 Mb interval on 3p21.3 by introducing truncated chromosome 3 (Abe et al. 2010). These results prove that human chromosome 3 carries factors, such as telomerase repressor gene(s), that control *hTERT* expression. LOH of the 3p21.3 region has been frequently observed in oral squamous cell carcinoma (OSCC) (Kasamatsu et al. 2011). We also showed that introducing human chromosome 3 into HSC3 cells, an OSCC cell line, suppressed *hTERT* transcription (Nishio et al. 2015); however, the 3p21.3 region possibly has the function of *hTERT* suppression in HSC3 cells.

Human artificial chromosomes (HAC), exogenous mini-chromosomes, are artificially created by chromosome engineering (Katoh et al. 2004). HAC vectors have several advantages as gene delivery vectors and are stably and independently maintained in host chromosomes. The capacity to carry large genomic loci with their regulatory elements allows the physiological regulation of the introduced gene like that of native chromosomes (Kazuki et al. 2011; Uno et al. 2018). In addition, HAC vectors can be transferred into any cell line throughout MMCT (Ohira et al. 2019a, b).

Previously, we reported the construction of HAC vectors containing the entire dystrophin genomic region (DYS-HAC) for gene therapy targeting Duchenne muscular dystrophy (DMD) (Kazuki et al. 2010). DMD is caused by the dysfunction of the dystrophin gene, such as a large deletion in the gene. Notably, several vectors have been developed for gene therapy of DMD; however, because the genomic size of dystrophin is huge (2.4 Mb), episomal vectors containing this entire region have not been reported (Kazuki et al. 2010). Therefore, DYS-HAC was developed for potential applications in DMD gene therapy. Thus, HAC vectors are useful for addressing large genomic regions and analyzing their functions.

This study investigated whether telomerase regulatory factors such as *hTERT* suppressor gene(s) were retained in the 3p21.3 region and could be involved in developing OSCC. Our results can provide important information regarding the functional significance of the LOH at the 3p21.3 region in OSSC. They may lead to identifying novel *hTERT* suppression genes encoded by the 3p21.3 locus.

## Materials and methods

### Cell culture

Chicken DT40 cells were obtained from Takeda (Kyoto University, Tokyo, Japan). A9, HSC3, and Hprt-deficient CHO (hprt − / −) cells were obtained from the Japanese Collection of Research Bioresources Cell Bank (Osaka, Japan). CHO K1 cells were obtained from ATCC (Manassas, VA, USA). Chicken DT40 cells containing truncated human chromosome 3 were cultured in RPMI medium 1640 (Invitrogen, Carlsbad, CA, USA) supplemented with 10% fetal bovine serum (FBS; HyClone, Logan, UT, USA), 1% chicken serum, 50 µM 2-mercaptoethanol, and 1.5 mg/mL G418 (Calbiochem, La Jolla, CA, USA) at 40 °C in a humidified incubator with 5% CO_2_. CHO K1 cells containing truncated chromosome 3 were cultured in Ham’s F-12 nutrient mixture (Invitrogen) supplemented with 10% FBS and 800 μg/ml G418 at 37 °C in a humidified incubator with 5% CO_2_. CHO (hprt − / −) cells containing HAC were cultured in a Ham’s F-12 nutrient mixture (Invitrogen) supplemented with 10% FBS and 8 μg/ml blasticidin S hydrochloride (Wako, Tokyo, Japan) at 37 °C in a humidified incubator with 5% CO_2_, as previously described. A9 and HSC3 cells containing a truncated chromosome 3 were cultured in Dulbecco’s Modified Eagle Medium (DMEM; Sigma, St. Louis, MO, USA) supplemented with 10% FBS and G418 (A9; 800 μg/ml, HSC3; 300 μg/ml) at 37 °C in a humidified incubator with 5% CO_2_. A9 and HSC3 cells were cultured containing a HAC vector in DMEM supplemented with 10% FBS and blasticidin S hydrochloride (A9, 8 μg/ml; HSC3; 3 μg/ml) at 37 °C in a humidified incubator with 5% CO_2_. All cell lines were confirmed to be mycoplasma-free using a MycoAlert Mycoplasma Detection Kit (Lonza, Walkersville, MD, USA) and were not passaged more than 20 times from the validated stocks.

### Plasmid construction

The 3p21.3-loxP targeting vector was constructed as an X3.2 vector, which contains additional restriction enzyme sites (SpeI, BamHI, PacI, and SacI) of the AgeI site in the X3.1 vector (Kononenko et al. 2013), which contains a loxP site and a 3′ HPRT site. X3.2 vector constructed the annealing oligo 5′-CCGGTACTAGTCGGGATCCCCTTAATTAAGGGGAGCTCA-3′ and 5′-CCGGTGAGCTCCCCTTAATTAAGGGGATCCCGACTAGTA-3′ into subcloned into AgeI sites of the X3.1 vector. For the addition of the sgRNA target site in X3.2, the annealing oligo 5′-TAACCAAACACGTACGCGTACGATGCTAGCT-3′ and 5′-AGCATCGTACGCGTACGTGTTTGGTTAAT-3′ subcloned into PacI/SacI sites of the X3.2 vector (X3.3). For the addition of the marker gene, the blasticidin S-resistant gene was amplified using PCR with the pEBmulti-Bsd vector (Wako) as a template and primers (5′-GGAAGATCTCCAGCAGGCAGAAGTATGCAAAGCA-3′ and 5′-GGACTAGTCAAGTTTCGAGGTCGAGTGTCAGTC-3′), digested with BglII/SpeI (NEB, Hertfordshire, UK), and cloned into the X3.3 vector (3p21.3-loxP targeting vector).

### CRISPR/Cas9 vector design

The CRISPR/Cas9 vector construction was performed as previously described (Uno et al. 2017). The CRISPR/Cas9 targeted sequences were designed using the website PITCh Designer 2.0 (https://www.mls.sci.hiroshima-u.ac.jp/smg/PITChdesigner/index.html). The DNA oligomer was inserted into the BbsI site of pX330-U6-Chimeric_BB-CBh-hSpCas9, which was a gift from Feng Zhang (Massachusetts Institute of Technology) (Addgene plasmid # 42,230).sgRNA target1 (D3S1568) targeting 5′-GATTGAGCGGCCCTCACTTCAGG-3′ was constructed with 5′-caccGATTGAGCGGCCCTCACTTC-3′ and 5′-aaacGAAGTGAGGGCCGCTCAATC-3′ primers. sgRNA Target 2, targeting 5′-GCATCGTACGCGTACGTGTTTGG-3′ was constructed with 5′-caccGCATCGTACGCGTACGTGTT-3′ and 5′-aaacAACACGTACGCGTACGATGC-3′.

### Transfection

The CHO hybrids (4 × 10^5^ cells) containing the truncated chromosome 3 were transfected with the loxP vector and Cas9 vectors by lipofection with a 0.2 µg loxP plasmid, and each of 0.8 µg Cas9 vector with 7.5 µl Lipofectamine 2000 reagent (Invitrogen) according to the manufacturer’s instructions. After 24 h in a basic growth medium, the cells were cultured in a medium containing G418. Fourteen days later, drug-resistant colonies were selected and expanded for further analysis.

CHO hybrids (4 × 10^5^ cells) containing HAC were transfected with the Cre expression vector pBS185 CMV-Cre by lipofection with 2 µg loxP plasmid with 7.5 µl Lipofectamine 2000 reagent (Invitrogen) according to the manufacturer’s instructions. After 24 h in a basic growth medium, the cells were cultured in a HAT (Sigma) medium. Fourteen days later, drug-resistant colonies were selected and expanded for further analysis.

#### MMCT

MMCT was performed as previously described (Ohira et al. 2019a, b). Briefly, donor cells were incubated with 0.05 μg/ml colcemid (Sigma) in F12 (Invitrogen) containing 20% FBS (JRH Biosciences) for 72 h. Micronuclei were harvested by treatment with 10 μg/ml cytochalasin B (Sigma) and centrifugation and sequentially filtered through 8, 5, and 3 μm polycarbonate filters (Whatman Nuclepore, Kent, UK). The fusion was mediated by 47% polyethylene glycol 1000 (Wako), followed by extensive washing with serum-free DMEM (Sigma). After incubation for 24 h in the culture medium, cells with chromosome transfer were selected with G418 (Sigma) or blasticidin S hydrochloride (Wako).

#### FISH

FISH was performed as previously described (Ohira et al. 2019a, b). Briefly, FISH analyses were performed in fixed metaphases of microcell hybrids using digoxigenin-labeled (Roche, Basel, Switzerland) human Cot-1 DNA (Life Technologies, Carlsbad, CA, USA) and biotin-labeled loxP, PAC (3p21.3 probe: RP6-234N4), or BAC (3q26.2 probe: RP11-82C9) vectors. The preparation of chromosomes, probes, hybridization, washing, and signal detection was performed according to our previous report. Briefly, chromosomal DNA was counterstained with DAPI (Sigma). Images were captured using an AxioImagerZ2 fluorescence microscope (Carl Zeiss GmbH, Jena, Germany) and analyzed using the Ikaros software program (MetaSystems, Altlussheim, Germany).

#### PCR

Genomic DNA was extracted from the cells using a standard method (Katoh et al. 2004; Kazuki et al. 2011). Primer pair #1 was: forward 5′-AGCAGACCCTGGCTACTCTT-3′ and reverse 5′-CGGAAATGAGAACAGGGGCA-3′.

Primer pair #2 was forward 5′-GCAGAAGGTACCCTAGACCG-3′ and reverse 5′-AGGTTCTCTGCCTGGGTTCT-3′.

Primer pair #3 was forward 5′-AGCAGACCCTGGCTACTCTT-3′ and reverse 5′-AGGTTCTCTGCCTGGGTTCT-3′.

Primer pair #4 was forward 5′-GGGCTGAATCACATGAAGGC-3′ and reverse 5′-CCCCTTGACCCAGAAATTCCA-3′.

Sequence-Tagged-Site (STS) Marker sequences are described in Supplementary Table S1.

### qRT-PCR

RNA isolation and reverse transcriptase (RT)-PCR were performed as described previously (Ohira et al. 2015; Ohira et al. 2019a, b). The mRNA expression of *hTERT* was analyzed using the following primers: forward, 5′-GCCTTCAAGAGCCACGTC, reverse: 5′-CCACGAACTGTCGCATGT. cDNA was amplified using an Applied Biosystems StepOne thermal cycler system and a SYBR Green PCR kit (Foster City, CA, USA). The mRNA levels were normalized against GAPDH mRNA (forward: 5′-AGCCACATCGCTCAGACAC, reverse: 5′-GCCCAATACGACCAAATCC).

### Statistical analysis

Data from more than three separate experiments are presented as mean ± S.D. Significance was established at *P* < 0.05, using an unpaired two-tailed Student’s *t*-test.

## Results

### Targeting of the loxP site in the 3p21.3 locus of the human truncated chromosome 3

**Fig. 1:**
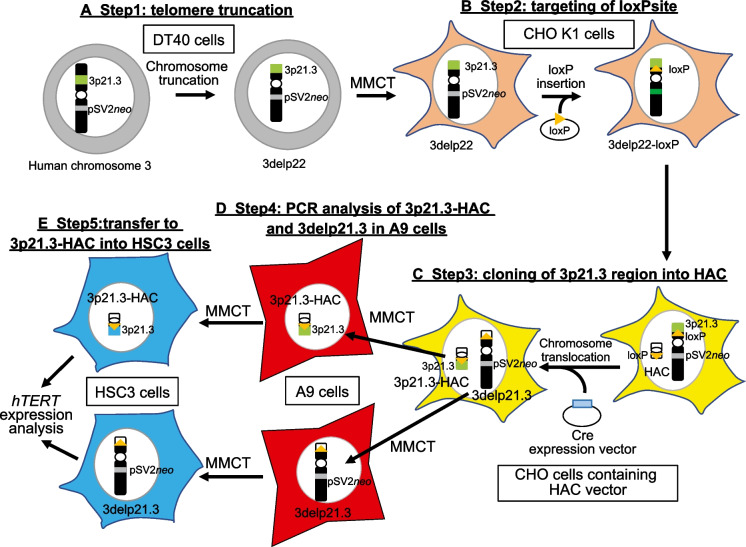
A schematic diagram of the construction of 3p21.3-HAC and establishment of HSC3 cells containing 3p21.3-HAC. **A** Human chromosome 3 (hChr.3) was modified by telomere-associated chromosomal truncation in DT40 cells. It was carried out at the telomere side of the 3p22 locus (3delp22). **B** Modified hChr.3 was transferred from DT40 to CHO-K1 cells via MMCT and inserted at the loxP site by CRISPR/Cas9 (3delp22-loxP). **C** 3delp22-loxP was transferred to CHO cells containing the HAC vector via MMCT. Cre/loxP-mediated reciprocal translocation generated the 3p21.3-HAC and a by-product (3delp21.3). **D** Chromosome transfer into A9 cells to separate the 3p21.3-HAC and 3delp21.3 by MMCT. **E** 3p21.3-HAC and 3delp21.3 were transferred to HSC3 cells via A9 cells

To investigate the gene(s) involved in regulating the expression of the *hTERT* gene within the 3p21.3 region in OSSC cells, we generated HACs with only the 3p21.3-p22.2 region (3p21.3-HAC) and truncated chromosomes that have lost this region and performed functional analysis using those chromosomes. The overall strategy for this approach is shown in Fig. 1.

**Fig. 2:**
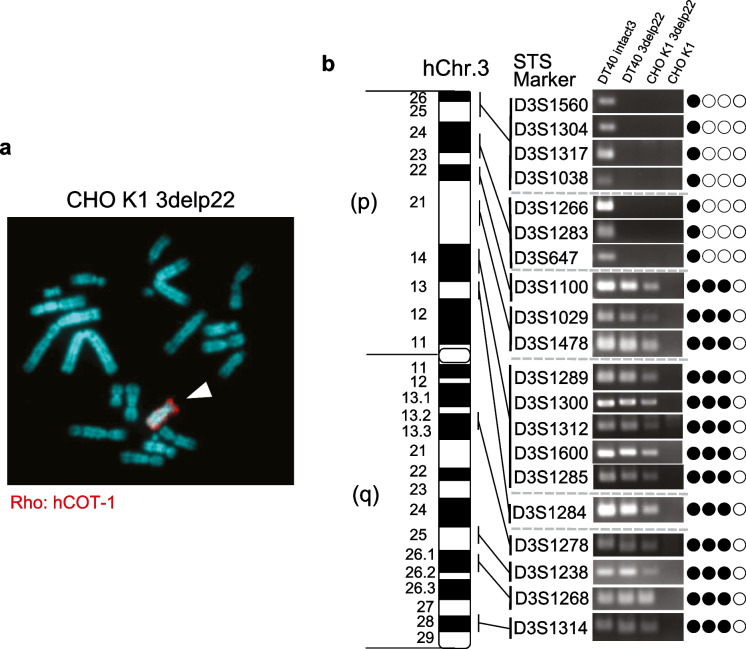
Establishment of CHO-K1 cells containing modified hChr.3 via MMCT. **A** FISH analysis with a digoxigenin-labeled hCot-1 DNA (red) identified the modified hChr.3 (3delp22) in CHO K1 cells. The arrowhead indicates the 3delp22. **B** Summary of PCR analyses on truncated p22 cells. Twenty STS markers on chromosome 3 examined were shown. Solid and open circles represent the presence and absence of truncated alleles at the tested loci in CHO K1 cells

HAC can clone megabase-sized genomic regions using the Cre/loxP-mediated gene cloning system (Kazuki et al. 2013). To clone the 7 Mb of the 3p21.3 region on human chromosome 3 (hChr.3), we used truncated hChr.3, which was deleted from the 3p22 locus to the telomere region of the chromosome (3delp22). This truncated chromosome was previously established using chicken DT40 pre-B cells containing neo-tagged hChr.3 (Abe et al. 2010) (Fig. 1, step 1). To insert a loxP site onto 3delp22 by CRISPR/Cas9, we transferred DT40 cells containing 3delp22 into CHO K1 cells, which are well-known for plasmid vector transfection efficiency, using microcell-mediated chromosome transfer (MMCT) (Fig. 1: step 2). As a result, the rearranged human chromosome was detected in CHO K1 cells using fluorescence in situ hybridization (FISH) analysis (Fig. 2A). To clarify the state of the rearranged human chromosome, we performed PCR analysis using 20 sequence-tagged site (STS) markers located on chromosome 3 (Fig. 2B). DT40 cells containing an intact human chromosome 3 were detected using PCR with all STS markers; however, DT40 and CHO K1 cells containing 3delp22 were not detected in the genomic region on the telomere side from 3p22. These results suggest that 3delp22 was successfully transferred from DT40 into CHO K1 cells.

**Fig. 3:**
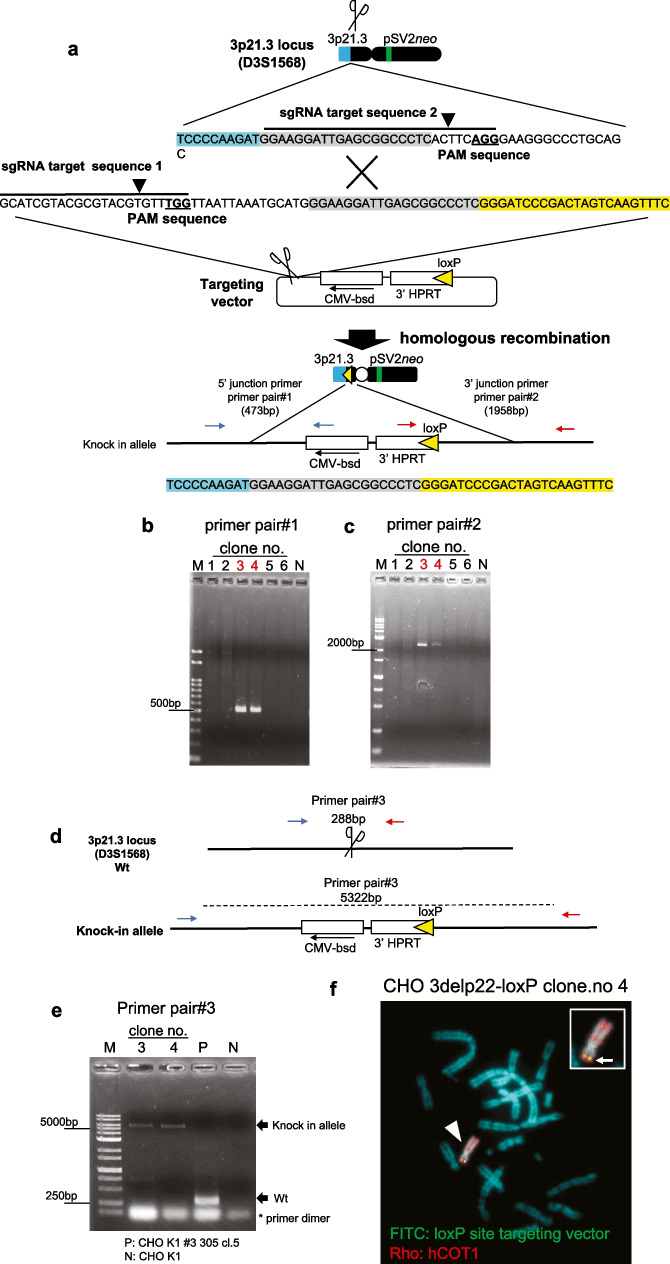
Targeting of loxP site on 3delp22 using CRISPR/Cas9. **A** A schematic outline of plasmid insertion encoding the loxP site into 3delp22. CRISPR/Cas9 targeting site was designed in the 3p21.3 region (sgRNA target 1) and the loxP site targeting vector (sgRNA target 2). A circular targeting vector was constructed with encoding 3′ region of the HPRT gene, the blasticidin-resistance gene, and the loxP site targeting the HAC vector shown. Primer pairs #1 and 2 were designed to detect the occurrence of homologous recombination. **B** and **C** The site-specific insertion event was detected in blasticidin-resistant clones using genomic PCR analysis (red for positive clones). The negative control (N) was CHO K1 cells. M indicates DNA markers. B shows the result of PCR using primer pair #1. C shows the PCR result using primer pair #2. **D** Schematic diagram of wild-type (Wt) allele of the 3p21.3 region and knock-in allele after plasmid insertion encoding the loxP site into the modified Chr.3. Scissors show the insertion site of targeting vector. Primer pair #3 was designed to distinguish between Wt and knock-in alleles by the size of PCR products. **E** PCR analysis using primer pair #3 shows the knock-in specific PCR product in CHO K1 clones targeting the loxP site (clone.3 and clone.4). The parental cell (P) was CHO K1 containing 3del22. The negative control (N) was CHO K1 cells. **F** FISH images after knock-in of loxP site in CHO K1 clones. Two-color FISH analysis was performed with digoxigenin-labeled hCOT-1 DNA (red) and biotin-labeled loxP site targeting plasmid vector (green). The arrowhead indicates the 3delp22-loxP. The inset shows enlarged images of the 3delp22-loxP (loxP site-specific probe-green signals are indicated by the arrow)

Next, we constructed a targeting plasmid vector to insert a loxP site into the 3p21.3 region using CRISPR/Cas9. The loxP site-targeting plasmid vector contained the 3’HPRT-loxP site, a CRISP/Cas9 target sequence (sgRNA target 1), and blasticidin S (*bsd*) gene for a positive selection resistance gene (Fig. 3A). The 3′ HPRT-loxP site is necessary to load a cloned genomic locus into the HAC vector using the Cre/loxP system. To increase the efficiency of homologous recombination using CRISP/Cas9, the target sequence (sgRNA target 1) contained a loxP-targeting vector (Fig. 3A). A PAM sequence serves as a binding signal for the Cas9 protein. CHO K1 cells containing 3delp22 were transfected with two CRISPR/Cas9 vectors, D3S1568 (for sgRNA target sequence 1) and sgRNA target plasmid vector (for sgRNA target sequence 2), and the loxP site targeting vector plasmid (Fig. 1: Step 2) to insert the loxP site into 3delp22. We established six of *bsd* resistance clones. Two (33%) clones (clone 3 and 4) showed the expected homologous recombination bands (product size of 473 bp and 1958 bp) by detecting specific primers for the 5′ junction sequence (primer pair#1) (Fig. 3B) and 3′ junction (primer pair#2) using PCR analysis with genomic DNA derived from these clones (Fig. 3C). A more detailed analysis of PCR using primer pair#3 (primer pair#1 forward primer and primer pair#2 reverse primer; product size of 5322 bp) showed that the loxP targeting vector was inserted into the D3S1568 (3p21.3) locus in both clones (clone 3 and 4) (Fig. 3D and E). Furthermore, the loxP site signal was observed on 3delp22 in CHO K1 hybrid cells (designated 3delp22-loxP) using FISH analysis (Fig. 3F). These results indicated that the loxP site was successfully inserted into the 3p21.3 locus (D3S1568) in the 3delp22.

### Construction of 3p21.3-HAC vector

**Fig. 4:**
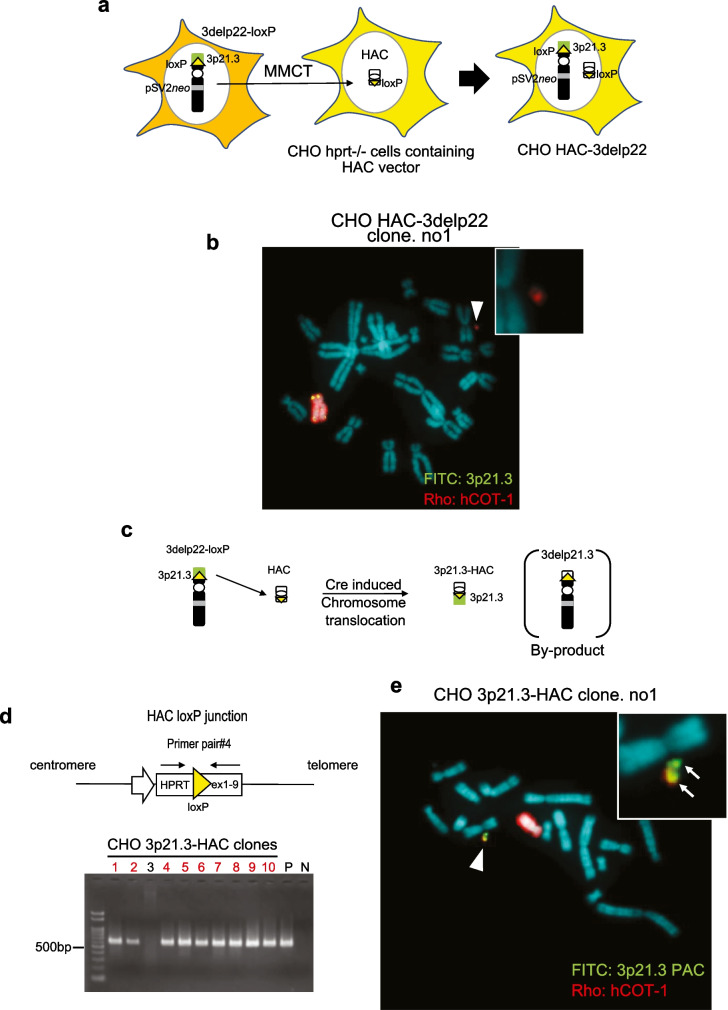
Cloning of 3p21.3-p22 locus into HAC vector. **A** A schematic diagram of 3delp-loxP transferring into CHO containing HAC. **B** FISH analysis of CHO cells containing the 3delp22-loxP and the HAC. Two-color FISH analysis was performed with digoxigenin-labeled hCOT-1 DNA (red) and a biotin-labeled PAC vector containing 3p21.3 genomic DNA region (green). The arrowhead indicates the HAC. **C** A schematic diagram of 3p21.3-HAC construction. The 3p21.3-p22 region (shown blue color) was cloned into the HAC vector via a Cre/loxP-mediated reciprocal translocation cloning system. **D** The site-specific insertion event was detected in HAT-resistant clones using genomic PCR analysis. A primer was designed to span the HAC insertion junction-specific site (indicated by a thin arrowhead in the upper); red clone number for positive clones. The positive control (P) was a CHO clone containing HSV TK-HAC, as previously described (Kazuki et al. 2011). The negative control (N) was CHO K1 cells containing HAC. **E** Fish analysis of CHO cells containing the 3p21.3-HAC and by-product (3delp21.3). Two-color FISH analysis was performed with digoxigenin-labeled hCOT-1 DNA (red) and a biotin-labeled PAC vector containing 3p21.3 genomic DNA region (green). The inset shows enlarged images of the 3p21.3-HAC (3p21.3 locus-specific probe-green signals are indicated by the arrow)

To clone the 3p21.3-p22.2 locus in 3delp22 onto a HAC (HAC2) (Kazuki and Oshimura 2011; Kazuki et al. 2011), the truncated chromosome (3delp22-loxP) in which the loxP sequence was inserted on the 3delp22 was transferred from CHO K1 cells to CHO hprt-/- cells containing the HAC vector using MMCT (Fig. 1, step 3; Fig. 4A). Forty-eight resistant CHO clones were isolated by G418 drug selection. These isolated G418 resistant CHO clones were confirmed by FISH analysis to retain both HAC and 3delp22-loxP (CHO HAC-3delp22) (Fig. 4B). To induce reciprocal translocation between the HAC and 3delp22-loxP chromosome, Cre expression vectors were transfected into CHO HAC-3delp22 cells (Fig. 4C), and we isolated ten resistant clones which were isolated in medium containing hypoxanthine, aminopterin, and thymidine (HAT). These clones were analyzed using PCR with HPRT reconstitution-specific primers and FISH with specific probes to detect the 3p21 region and HAC DNA. Nine out of ten drug-resistant clones were detected as PCR products using HPRT-reconstitution-specific primers (primer pair#4) (Fig. 4D). FISH analysis indicated that the 3p21.3 region was inserted into the HAC in two out of nine clones (Fig. 4E).

**Fig. 5:**
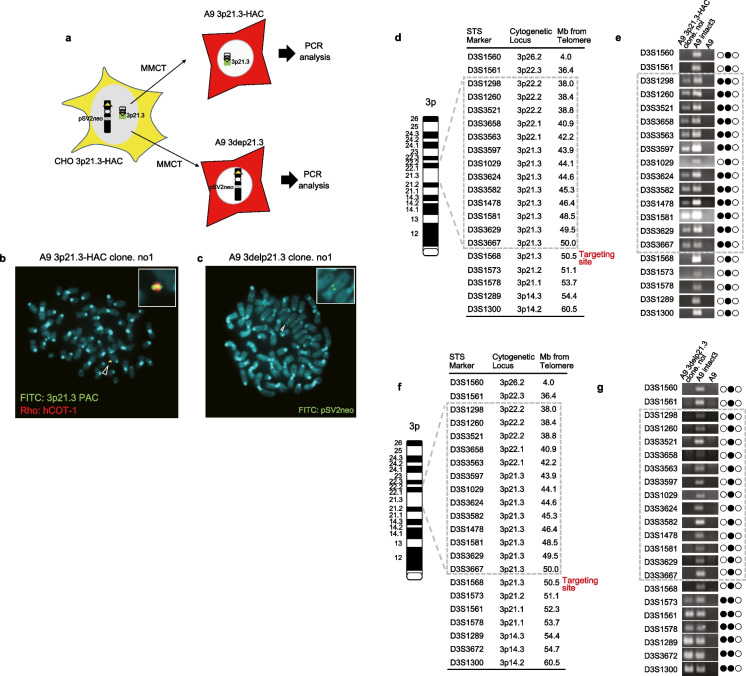
Characterization of 3p21.3-HAC and 3delp21.3. **A** A schematic outline of the separation of 3p21.3-HAC and 3dep21.3. **B** FISH analysis with a digoxigenin-labeled hCot-1 DNA (red) and a biotin-labeled PAC vector containing 3p21.3 genomic DNA region (green) identified 3p21.3-HAC in A9 cells. **C** FISH analysis with a biotin-labeled psV2*neo* plasmid vector (red) identified 3delp21.3 in A9 cells. **D** Summary of using STS marker in PCR analyses on A9 3p21.3-HAC cells. D3S1568 was the targeting site for loxP insertion. **E** Twenty STS markers on the chromosome 3p arm examined were shown. Solid and open circles represent the presence and absence of the 3p arm locus at tested loci in A9 3p21.3-HAC cells. **F** Summary of using STS maker in PCR analyses on A9 3delp21.3 cells. **G** Twenty STS markers on the chromosome 3p arm examined are shown. Solid and open circles represent the presence and absence of the 3p arm at tested loci in A9 3delp21.3 cells

These drug-resistant clones carried two chromosomes: 3p21.3-HAC and 3delp21.3 (Fig. 4D). Therefore, we could not confirm whether the 3p21.3 region was a clone of the HAC vector. We first performed chromosome transfer into A9 cells to separate 3p21.3-HAC and 3delp21.3 using MMCT (Fig. 1, step 4; Fig. 5A). We established seven bsr-resistant A9 3p21.3-HAC clones and eight G418-resistant A9 3delp21.3 clones. The signal of the 3p21.3 probe on the HAC vector in three of the seven A9 3p21.3-HAC clones was detected using FISH analysis (Fig. 5B). Furthermore, the pSV2*neo* probe signal on human chromosome fragments was observed in one of eight A9 3delp21.3 clones (Fig. 5C). Moreover, on the one hand, PCR analysis using 20 STS marker sets detected genomic loci from 3p22.2(D3S1298) to 3p21.3 (D3S3667) in A9 3p21.3-HAC clones; however, it did not detect the telomere side from 3p22.2 and centromere side from 3p21.3 (Fig. 5D and E). On the other hand, A9 cells containing 3delp21.3 were not detected in the 3p22.2-3p21.3 locus by PCR using STS markers (Fig. 5F and G). These results indicated that the 3p22-3p21.3 locus was successfully inserted into the HAC vector.

### 3p21.3-HAC suppresses hTERT transcription in HSC3 cells

**Fig. 6:**
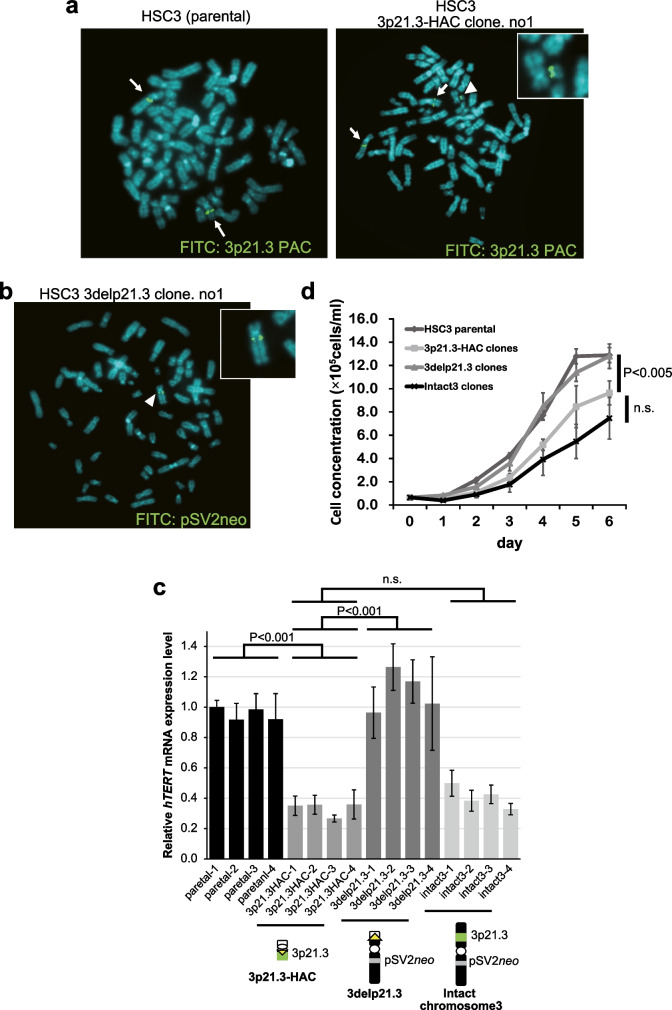
*hTERT* suppression effect of 3p21.3-HAC in HSC3 cells. **A**FISH analysis with a biotin-labeled PAC vector containing 3p21.3 genomic DNA region (green) identified parental HSC3 cells (left panel) and HSC3 3p21.3-HAC cells (right panel). The arrow shows endogenous 3p21.3 genomic region in the HSC3 cells. The arrowhead indicates the 3p21.3-HAC in the HSC3 3p21.3-HAC clone. The inset shows enlarged images of 3p21.3-HAC. **B** FISH analysis with a biotin-labeled pSV2*neo* plasmid vector (green: modified h.Chr3 specific probe) identified HSC3 3delp21.3 clone. The arrowhead indicates the 3delp21.3. The inset shows enlarged images of 3delp21.3. **C**. qRT-PCR analysis of relative *hTERT* RNA expression levels in parental cells, HSC3 3p21.3-HAC clones, HSC3 3delp21.3 clones, and HSC3 containing intact hChr.3 clones. Expression in one of the parental cells was arbitrarily set at 1. *GAPDH* mRNA expression was used as the internal control. Data are presented as means ± S.D. of three independent experiments (*P* < 0.001, ns: not significant). **D** Cell number of parental cells, HSC3 3p21.3-HAC clones, HSC3 3delp21.3 clones, and HSC3 containing intact hChr.3 clones. The bars correspond to means ± S.D. of three independent experiments (*P* < 0.005, ns, not significant)

To determine whether 3p21.3-HAC encodes *hTERT* suppression genes, we transferred 3p21.3-HAC from A9 to HSC3 cells using MMCT (Fig. 1, step 5). The presence of the 3p21.3-HAC in the HSC3 microcell hybrid clone was clarified using FISH analysis with a PAC probe containing genomic DNA from the 3p21.3 region. The 3p21.3 probe was detected on a mini chromosome and two copies of endogenous chromosome 3 in HSC3 microcell hybrid clones with 3p21.3-HAC (Fig. 6A). As a negative control, we used 3delp21.3 tagged with pSV2*neo* that resulted from the establishment of 3p21.3-HAC (Fig. 1, step 3; Fig. 4D) in CHO cells. This 3delp21.3 was also transferred from A9 to HSC3 cells using MMCT. The presence of 3delp21.3 with the pSV*neo* probe was detected in HSC3 microcell hybrid clones (Fig. 6B). To determine the copy number of 3p21.3 region in parental cells and microcell hybrid clones, we performed an additional FISH analysis. In most parental and 3delp21.3 clones, two copies of the 3p21.3 region were observed, while three copies were observed in 3p21.3-HAC clones (Supplementary Table S2). This indicates that in most cases, one copy of the HAC was transferred in the 3p21.3-HAC clones.

To investigate *hTERT* transcription in HSC3 microcell hybrid clones carrying 3p21.3-HAC or 3delp21.3, we performed qRT-PCR analysis. HSC3 microcell hybrid clones with an introduced 3p21.3-HAC exhibited suppressive effects on *hTERT* transcription, which was similar to the HSC3 microcell hybrid clones with intact chromosome 3 (reduced *hTERT* transcription to 64–73% of control cells with parental cells) (Fig. 6C). However, HSC3 microcell hybrid clones with 3delp21.3 were similar to those of parental cells with high *hTERT* transcription. Therefore, these results prove that the 3p21.3-p22.2 region carries *hTERT*-related suppressor gene(s) in HSC cells. To further investigate whether suppression of *hTERT* transcription affects the proliferative rate, we performed a comparative analysis of the proliferation rate of the parental cells, 3p21.3-HAC clones, 3delp21.3 clones, and intact3 clones. The results showed that the 3p21.3-HAC clones showed a significantly decreased growth rate compared with the parental cells and 3delp21.3 clones. In addition, compared with intact3 and 3p21.3-HAC, there was a tendency for an even lower proliferative rate in intact3; however, the difference was insignificant. These results suggest that the suppression of *hTERT* expression by 3p21.3-HAC negatively regulates cell proliferation.

## Discussion

We previously showed that the *hTERT* suppressor gene encodes human chromosome 3 in RCC and OSCC cell lines (Abe et al. 2010; Nishio et al. 2015). In addition, we identified *hTERT* repressor genes within the human chromosome 3p21.3 region via functional analysis of various truncated chromosome 3 fragments in RCC cell lines (Abe et al. 2010). This study demonstrated that cloning the 3p21.3-p22.2 region into the HAC vector (3p21.3-HAC) by chromosome engineering and 3p21.3-HAC repressed *hTERT* mRNA transcription in HSC3 cells. These data suggest that only the 3p21.3 region has the suppression of the *hTERT* expression effect and that the other chromosome 3 region are not involved in the regulation of *hTERT* mRNA transcription.

Loss of the 3p21.3 region is frequently found in kidney, oral, and other cancers (Hesson et al. 2007). Furthermore, some genes have been identified as tumor suppressors in this region. Ras-associated domain family protein 1A (*RASSF1A*) is a tumor suppressor gene in the 3p21.3 region. Overexpression of *RASSF1* induces cell cycle arrest in lung carcinoma cell line H1299. *RASSF1A* inhibits cell cycle progression by preventing the accumulation of Cyclin D1 (Shivakumar et al. 2002). *Rassf1a* KO mice spontaneously develop lung cancer, lymphoma, and breast cancer (Tommasi et al. 2005). In addition, Zinc finger MYND domain-containing protein 10 (*ZMYND10*) is a tumor suppressor gene in the 3p21.3 region. Ectopic expression of *ZMYND10* in breast cancer cells induces apoptosis and inhibits cell growth, migration, and invasion in vitro and in vivo. *ZMYND10* suppresses *NEDD9*, a known oncogene related to metastasis and mRNA translation, by upregulating miR-145-5p (Wang et al. 2019). However, no gene(s) has(ve) yet been found to regulate *hTERT* transcription in the 3p21.3 region directly.

Non-coding RNAs may potentially affect the transcriptional regulation of *hTERT*. Long intergenic non-coding RNAs (lincRNAs) are long (> 200 bp) non-coding RNAs that do not overlap regions of protein-coding and are categorized as long non-coding RNAs (lncRNAs) (Ulitsky and Bartel 2013). Notably, some research groups have shown that lincRNAs are important regulators of multiple cellular and molecular processes, such as chromatin modification and transcriptional regulation (Ransohoff et al. 2018). Moreover, lincRNAs are associated with cancer development (Bhan et al. 2017; Peng et al. 2017). For example, lincRNA-p21 exerts its tumor-suppressive effect in OSCC cell lines by inhibiting the JAK/STAT signaling pathway, resulting in the induction of apoptosis (Jin et al. 2019). In addition, enhancer RNAs (eRNA), which represent a class of relatively lncRNAs (50–2000 bp) transcribed from the DNA sequence of enhancer regions, play important roles in cellular processes during development and diseases, including cancer (Wang et al. 2020). One of the eRNAs, the p53-bound enhancer regions (p53BERs), produces eRNAs in a p53-dependent manner. eRNAs act as gene enhancers and interact intrachromosomally with multiple neighboring genes to convey long-distance p53-dependent transcriptional regulation (Léveillé et al. 2015; Melo et al. 2013). Methylation-dependent inactivation of the eRNA promoter has been observed in various human cancers (Léveillé et al. 2015). These examples suggest the importance of lncRNA gene regulatory functions in tumor suppressor signaling pathways. Intriguingly, more than 10 long non-coding RNAs (lncRNAs) with unknown functions have been found in 3p21.3 (UCSC Genome Browser web tool. https://genome.ucsc.edu/). This indicates that lncRNAs, including lincRNAs, may be involved in regulating *hTERT*.

MicroRNAs (miRNAs) are non-coding RNAs. The overexpression of miR-138, coded on the 3p21.3 region, was reported to downregulate *hTERT* protein levels but not *hTERT* mRNA in human cervical cancer and thyroid carcinoma cell lines (Mitomo et al. 2008). In contrast, miR-138 is significantly upregulated in OSCC tumor tissues compared to normal tissues near the tumor (Zhang et al. 2017). Therefore, it is unlikely that miR-138 functions as a transcriptional repressor of *hTERT* in OSSC. Moreover, miRNAs coding for the 3p21.3 region have been reported to have tumor-suppressive functions. miR-191 is down-regulated in OOSC tissues (Gissi et al. 2018). Overexpression of miR-191 can block the migration and proliferation of lung cancer cells by downregulating the Wnt signaling pathway (Zhou et al. 2020). miR-771 inhibits gastric cancer cell proliferation by downregulating CDK4 expression (Liao et al. 2016). Interestingly, both miR-771 and *RASSF1* are located in the 3p21.3 region, and miR-771 is upregulated by *RASSF1A* (Liao et al. 2016). This phenomenon suggests that the genes in the 3p21.3 region may function as specific chromosomal domains involved in cancer suppression.

Human cytochrome P450 family 3 subfamily A (*CYP3A*) is the most abundant P450 isozyme in the human liver and small intestine, metabolizing approximately 50% of commercially available medical drugs. Using the HAC vector system, we cloned approximately 700 kb of the human *CYP3A* gene cluster (*CYP3A4*, *CYP3A5*, *CYP3A7*, and *CYP3A43*) containing its regulatory region into the HAC vector and generated mice transferred with the CYP3A-HAC vector. To generate fully *CYP3A* humanized mice (CYP3A-HAC/Cyp3a-KO), we also generated mice in which the endogenous mouse *Cyp3a* gene cluster was disrupted (Cyp3a-KO mice) and crossed them with CYP3A-HAC mice. In these mice, tissue-specific expression of *CYP3A4* was observed in the liver and small intestine, similar to that in humans. In addition, *CYP3A4*, specifically expressed in the adult stage, and *CYP3A7*, specifically expressed in the fetal stage, were expressed in the adult and fetal stages, respectively (Kazuki et al. 2013). These results suggest that the genes carried on the HAC vector function correctly under the epigenetic genetic control of the host.

Recent studies have revealed that topological associate domains (TADs) contribute to gene regulation by restricting chromatin interactions between target genes and regulatory sequences such as enhancers. TADs are the basic units of 3D nuclear organizations (van Steensel and Furlong 2019; Zheng and Xie 2019). TADs regulate gene expression by restricting the interactions between cis-regulatory sequences and target genes. Notably, most studies suggest that TADs regulate gene expression by restricting enhancer-promoter interactions to each TAD (Dixon et al. 2016). In mammals, TAD boundaries are usually demarcated by the chromatin architectural proteins CCCTC-binding factor (CTCF) and cohesin. CTCF is a DNA-binding protein that recognizes specific sequence motifs (van Steensel and Furlong 2019).

Interestingly, other groups have shown the potential of CpG sites essential for transcription of the *RASSF1A* and *ZMYND10* (also known as *BLU*) genes at the sequence level, suggesting that the transcriptional regulator CTCF binding to the insulator of the *ZMYND10* gene results in the formation of a barrier within the separate epigenetic domains of the *RASSF1A* and *ZMYND10* genes juxtaposed in the 3p21.3 gene cluster region (Chang et al. 2010). Therefore, CTCF binds to the insulator of the *ZMYND10* gene and exerts a barrier function in the separate epigenetic domains of the *RASSF1A* and *ZMYND10* genes juxtaposed in the 3p21.3 gene cluster region. These results suggest that TAD may be present in *ZMYND10* and *RASSF1A* in at least the 3p21.3 region. Moreover, there is strong evidence for a mechanism by which TADs regulate the expression of tumor suppressor genes in the 3p21.3 locus. In this study, we inserted the full-length region of the 3p21.3 region into the HAC vector. Thus, HAC-containing target genomic loci may reproduce the physiological genomic context, including TAD, for tumor suppressor gene expression. Further investigation involving identifying novel *hTERT* regulatory factors on 3p21.3-HAC using RNA sequence analysis and chromatin interaction analysis (the Hi-C method (Kempfer and Pombo 2020)) in HSC3 3p21.3-HAC clones may be of great help in understanding the molecular mechanism of *hTERT* reactivation in cancer cells.

## Supplementary Information

Below is the link to the electronic supplementary material.

Supplementary file1 (XLSX 14 KB)

Supplementary file2 (XLSX 13 KB)

## Data Availability

The datasets created and/or analyzed during the current study are available from the corresponding author upon reasonable request.

## Abbreviations

*CYP3A*: Cytochrome P450 family 3 subfamily A
*DMD*: Duchenne muscular dystrophy
*DYS-HAC*: HAC vectors containing the entire dystrophin genomic region
*FISH*: Fluorescence in situ hybridization
*HAC*: Human artificial chromosome
*hTERT*: Human telomerase reverse transcriptase
*LOH*: Loss of heterozygosity
*MMCT*: Microcell-mediated chromosome transfer
*OSCC*: Oral squamous cell carcinoma
*RCC*: Renal cell carcinoma
*STS*: Sequence-tagged site
*3p21.3-HAC*: HAC vector with only the 3p21.3-p22.2 region
*3delp21.3*: Truncated chromosome 3 with deletion of the 3p21.3 region
